# Dental Miscellanies

**Published:** 1872-08

**Authors:** H. Scott


### Dental Miscellanies.

BY H. SCOTT.

Oxychloride of zinc; what of it? Are those who have been experimenting with it a few years ready to report intelligently? I hear less of it lately. Have any found bony arches thrown over exposed pulps where the os artificial had been previously used, and the nerve and its membranes still living and healthy? Have opportunities for such observations occurred with sufficient frequency to form the predicate for reliable practice? The profession should know whatever is known to any of its members. The cases that have occurred to me, *post extractum*, have, without one exception, shown empty chambers that once were filled with living, healthy tissues; sometimes mummied, sometimes wreaking with effete matter. I believe, however, that where the oxychloride has been used for caping, it has generally brought about an easy and painless death; but death nevertheless.

For quieting sensitive dentine, where excavation has been difficult, this agent has been useful to me, but it has not occurred to me to know its *modus operandi*, whether that of a sedative merely, or, on the other hand, a destroyer of vitality by its causticity. Without knowing more than at present, however, I am obliged to record my fears of its devitalizing power. At least, with my present experience, I will not trust it in very young or immature teeth. Where the membrane is absolutely uncovered, or but the merest film of bony covering over it, I apprehend it will not matter what we do, the nerve will perish in more than ninety-five cases out of the hundred. In filling fangs, in some cases the os artificiel may, I believe, be used with very good satisfaction; beyond that it will certainly go out of use, as it is too unsubstantial for tooth filling. It is at once an escorotic and a foreign substance, and can not therefore, as I have before said, be tolerated in contact with or near proximity to the exquisitely sensitive and delicate tissues of tooth, nerve, and membrane. I would be glad to hear experiences in exchange for my own.

### Case No. 1.

Young C—— came three months ago to have some fillings made. It was his first visit of the kind, though I had been filling the teeth of his family for thirty years or more. He was at the time about twenty-two years of age. The left upper latteral incisor lapped the central a little, and was much decayed. In the preparation for filling, the nerve pulp was closely approximated. I used no non-conductor, because I did not know what to use, and had no confidence in any. I filled the tooth with gold under protest in my own mind, using no especial force immediately over the thin covering, so that I am quite sure no impingement of the membrane took place from depression of the bone. I directed the patient to call immediately if toothache set in. All was quiet during four or five weeks, but he came at last with his face frightfully swollen, the tooth very loose and exceedingly painful. He had disregarded my injunction by waiting twenty-four hours after the pain commenced. I removed the tooth and found every part of the investing membrane deeply inflamed. Would a non-conductor have saved the trouble that followed in this case? I have filled many teeth under the same circumstances, with no untoward results. The young man wanted the price of the filling deducted from his bill, as he had lost the tooth. This request I registered on the page of the pleasures of professional life reversed.

### Case No. 2.

Mr. G—— had toothache in the first left inferior molar. He was a man of thirty years, and at the time in good health. The body of the tooth was sound, and the involucre healthy. The arsenic was used as a devitalizer in the usual way, and remained six hours, when there were indications of peri-odontitis. I prescribed prudent conduct, but the symptoms increased with great rapidity, utterly defying all attempts to subdue or modify the inflammation, and at the end of about twenty-four hours we were obliged to extract the tooth. There was no swelling, but the alveolar and dental membranes were fearfully inflamed. I expected a speedy solution of the case after the removal of the tooth, but to my great surprise the pain not only continued but increased. I directed fomentations and saline aperients. In ten days the case was culminating by the formation of an abscess in the thick muscles of the cheek, the immediate surroundings of the extracted tooth having resumed a healthy condition. The abscess was pointing toward the external surface, and was, in violation of my special order, lanced on the outside, where there is left an unsightly scar. Nothing that I know of could have resolved this case, but why the inflammation was transferred to the previously healthy tissues of the cheek, is unexplained by pathology. Could the fomentations have had anything to do with the transfer?

CASE NO. 3.

A year ago I attempted to remove the second left lower molar from the mouth of Mr. P——. The tooth stood alone, and was very loose from absorption of the alveolar bones. One would have supposed that but trifling force would have been required to lift it away, and yet I expended all my strength with a strong pair of forceps three times, without making the slightest impression upon it. Two stronger men have since tried their muscle on it, but the molar is still in *situ*. I proposed to split the tooth, or penetrate to the nerve with a drill for the purpose of destroying it, but the patient objected. The tooth is not in the least decayed, but is the subject of attacks of violent toothache, and is always sensitive to variations of temperature. It is simply a "hard case."

What is to be the final practice on teeth having the complication of exposed nerves? Will caping ever succeed in a sufficient number of cases to justify its use as the rule? And can inflamed pulps be restored to health and subsequently be bridged over with new formations of bone, so as thereafter to remain in as good, or approximately good condition as those of other teeth filled before their nerves have become pathologically affected? Can any man say that such results ever take place? I believe all such expectations are futile, and destined to meet with disappointment. The reproduction of tooth bone is not to be expected. In other bones osseous deposits take place, as in the case of fractured bones, and the like. The ossification of teeth is from the rudimental pulp, and when once completed is done forever, because the conditions are destroyed with the fulfillment of their function. The projection of the fangs of teeth after the formation of the crowns is a different thing; but even fangs are never mended by the assimulating process. The formation of exostosis may not be named as an exception, since those growths are beyond all doubt of a morbid or pathological nature. Nature does, nevertheless, now and then perpetrate a freak outside of her established order; but such freaks are always strictly fortuitous, and very far between, and being thus, afford no basis for scientific or safe practice.

My experience up to this time does not allow one to depend upon caping exposed nerves with any assurance of good results. I am compelled to say that until more is known than at present, we must depend upon removing the nerves of such teeth, and then employ our best skill in treating and filling the fangs and crown cavities, beyond which all is to be trusted to the *vis medicatrix naturæ*. If we understood and could control the laws of vital chemistry, we should not be groping in the dark on this subject; as it is, we must do the best we can by dealing with what we know to be facts, and wait for the development of new facts. This is my status on exposed nerves, and although I am by no means too old to learn, I feel that the sooner we all come to this practice the sooner we shall stand on safe ground.

